# Implementation of a model of awareness-raising for taxi motorcyclists in Benin in relation to helmet use: protocol for a quasi-experimental study

**DOI:** 10.1186/s12889-021-10298-7

**Published:** 2021-01-28

**Authors:** Bella Hounkpe Dos Santos, Alphonse Kpozehouen, Yolaine Glele Ahanhanzo, Donatien Daddah, Edgard-Marius Ouendo, Alain Leveque, Yves Coppieters

**Affiliations:** 1 Ecole de Santé Publique, Université Libre de Bruxelles, Brussels, Belgium; 2 Institut Régional de Santé Publique, Ouidah, Benin

**Keywords:** Quasi-experimental, Awareness, Helmet, Traffic accident, Effectiveness

## Abstract

**Background:**

In the large cities of Benin, motorcycle taxi drivers, mainly between the ages of 20 and 40, are particularly exposed to accidents due to their profession. User awareness, along with legislative reforms and enforcement measures, would reduce the incidence of crashes and injuries. This study aims to test the effectiveness of an awareness-raising model regarding helmet use for motorcycle taxi drivers.

**Methods:**

This is a quasi-experimental study that will take place in the cities of Parakou (intervention group) and Porto Novo (control group). Over a three-month period, a package of awareness-raising activities will be implemented in the intervention area, targeting a group of motorcycle taxi drivers. The messages to be developed for awareness-raising will focus on the most frequently influencing factors, as identified by the baseline collection. These key messages will be disseminated through various tools and communication channels (banners, motorcycle stickers and motorcycle taxi uniforms, interactive sessions). Data will be collected prospectively via a self-reported questionnaire and observation, carried out before the intervention, at the end, and 6 months later. The data will relate to knowledge, attitudes and practices regarding helmet use. The analysis will compare the indicators between the groups, as well as between the pre- and post-intervention phase. The KoboCollect software will be used for data entry and processing, and Stata 15 will be used for data analysis. Chi-square or Fisher, Student’s or Kruskal-Wallis tests will be used for the comparisons. The difference-in-difference method will be used to determine the specific effect of the awareness activities.

**Discussion:**

This study will assess the contribution of awareness messages to changing the behaviour of motorcycle taxi drivers by determining the specific effect of the intervention.

## Background

Road accidents are a major public health problem across the world. They are the leading cause of death for young people aged 15 to 29. Apart from the high number of deaths in the economically active population, these accidents also cause disabilities and represent a heavy economic burden for families and countries. Low-income countries account for around 13% of road deaths [1]. In most African countries, the use of vehicles that do not meet key safety standards, the dilapidated state of road infrastructure, and the absence, inadequacy or insufficient enforcement of road safety laws continue to expose road users to fatal road accidents [2, 3]. Added to this are the behaviours of road users. One of the main risk factors for road accidents and related trauma is the attitudes and behaviours of users, most notably: speeding; driving under the influence of alcohol or any other psychoactive drug; not wearing a helmet, seatbelt or child restraint; and distracted driving, such as using a mobile phone [1, 4–8]. Despite these well-known factors, superstitious drivers are more likely to attribute accidents to fate [9]. Although aware of the protection offered by helmets, many motorcycle drivers and passengers do not wear one [10, 11]. This situation is all the more worrying, since the most vulnerable road users, such as pedestrians, cyclists and motorcyclists, account for more than half of all road deaths in the African sub-region, according to the World Health Organization (WHO). This figure is an underestimate, due to the poor quality of the data provided by the countries in the region, especially when we consider the rising number of motorcycles and journeys by motorcycle in these countries, which is contributing to the increase in road accidents [1, 12]. Accidents cause motorcyclists more limb injuries than head injuries, but the latter are responsible for almost half of all deaths [6]. In their meta-analysis, Liu et al. found that wearing helmets reduced the risk of head trauma and death [13]. Similarly, in his cross-sectional study, Singleton argues that skull fractures, brain contusions and intracranial haemorrhages were significantly less common among helmeted motorcyclists injured in road crashes than among those not wearing a helmet [14].

In Benin, young people aged 20 to 40 are the group most frequently involved in road accidents. They also account for nearly half of all victims injured or killed in such accidents. In addition, motorcycles are involved in more than half of all accidents, and their drivers or passengers represent more than half of the fatalities [15]. In Benin, motorcycles are the main means of travel for road users. The proportion of households that owns a motorcycle continues to grow, rising in 10 years from less than 45% in 2001 to more than 55% in 2011 [16]. Motorcycle taxi drivers are among those who travel mainly by motorcycle, using this means of transport as a taxi to carry passengers. This mode of transport is mostly used for trips within cities. These motorcycle taxi drivers do not always perceive the risks associated with their profession [17].

According to the WHO and several authors, in low- and middle-income countries, only an approach integrating user behaviour and several other interventions will be able to prevent trauma and death from road accidents in a cost-effective manner [1, 18–21]. The main effective interventions are legislative reforms accompanied by political will, and implementing measures [1, 18, 20], such as awareness-raising and education of the population [22], and increased police control [1, 19]. Concerning specifically the wearing of helmets, the implementation of helmet legislation seems to be effective in increasing the use of helmets, and reducing head injuries and deaths from road accidents [23–25], even more so if it is accompanied by public awareness and education, which affect user knowledge and attitudes towards helmet-wearing behaviour [22, 26]. User knowledge is defined as the state of knowing about helmet wearing, and attitude is understood as users’ subjective judgement, specifically their beliefs about the likely consequences of wearing a helmet [27]. To ensure behavioural change in individuals, it is necessary to implement educational interventions based on proven theories or models [26–28]. According to the theory of planned behaviour, behaviour is determined by intention, which is the conscious decision to take a certain action. It is guided by a combination of three considerations: attitude, the subjective standard, and the perception of control over behaviour. According to this theory, attitude is the set of people’s beliefs regarding the consequences of the said behaviour, multiplied by the evaluation of those consequences. These are the judgments about the desirability of the behaviour and its consequences. The subjective standard is an individual’s set of normative beliefs, and his or her motivation to comply with the standards. It is therefore the perceived social pressure to conform or not conform to the behaviour, the considerations of influence, and the opinion of relatives on the behaviour. Perceived behavioural control is the perceived ease or difficulty of performing a given behaviour: the belief in one’s ability to succeed in the targeted behaviour. In addition, environmental, demographic and personal factors influence all three types of beliefs [26–29].

Benin adopted the law on compulsory helmet wearing for motorcycle drivers and passengers in April 1972, but it was not accompanied by enforcement measures. It was not until 2014 that this law began to be effectively implemented for motorcycle drivers, with mass awareness-raising, police controls and penalties. It is clear, however, that there are still drivers who do not always wear helmets, especially in certain localities of the country. How effective would a helmet awareness programme for motorcycle taxi drivers in Benin be? Would such a programme help to reduce cases of road accident-related traumatic brain injury within this target group?

## Research hypothesis and objectives

### Hypothesis

Raising awareness among motorcycle taxi drivers on the need to respect the law and carrying out helmet checks, increases the use of helmets among these users, thereby helping to reduce the risk of traumatic brain injury within this target group.

### Main objective

Our study aims to test the effectiveness of an awareness model to improve the helmet-wearing behaviour of motorcycle taxi drivers and to help reduce the risk of traumatic brain injury among this target group.

### Specific objectives

The specific objectives of this study are:

✓ To determine the level of knowledge held by motorcycle taxi drivers on the role of helmets, in each of the two zones, at the start and at the end of the driver awareness programme.

✓ To compare the helmet-wearing attitudes of the two groups of motorcycle taxi drivers in the two study areas, at the start and at the end of the programme.

✓ To determine the proportion of motorcycle taxi drivers who always and correctly wear a helmet in each of the two zones, at the start and at the end of the programme.

✓ To compare the frequency of traumatic brain injury in the two groups.

## Methods

### Study framework

**Fig. 1:**
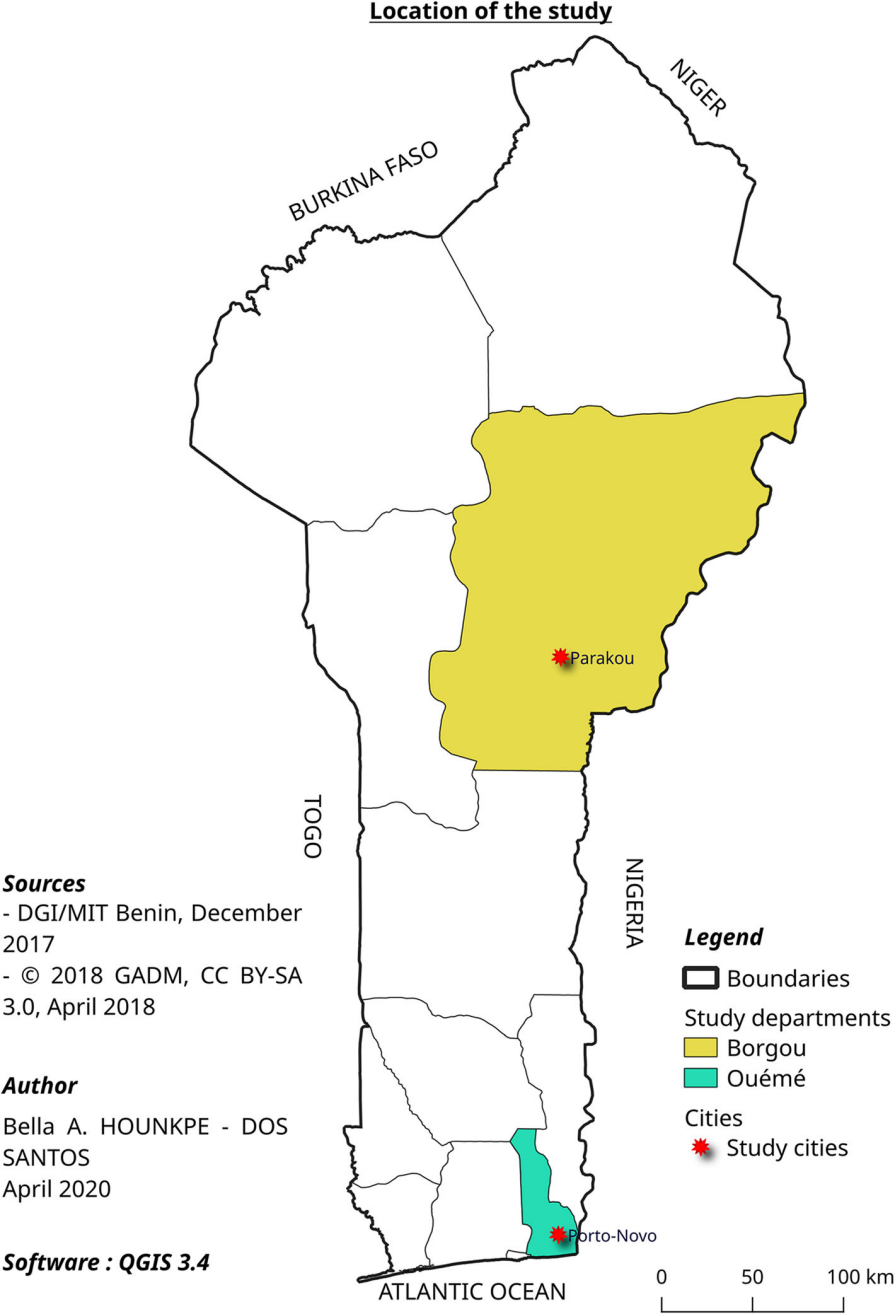
Location of the study. Departments and administrative boundaries of Benin. Study departments are marked in different colours. Red stars represent study cities. Data sources: DGI/MIT Benin and GADM.org. Copyright holder: BHDS

The study will take place in two cities in Benin: Porto Novo and Parakou. To identify the study cities, we took into account the fact that these two cities are the second (Porto Novo) and third (Parakou) largest in the country. In both cities, legislation concerning the wearing of helmets is enforced, but not always consistently. They are also located in departments that are at the two extremes of the country (north and south), reducing the risk of control group contamination (Fig. 1). In these two cities, as in the rest of the country, motorcycle taxi drivers are organised in unions of motorcycle taxi drivers. They have gathering places called motorcycle taxi drivers’ parks. These parks, known to customers, are often enclosures located on the edge of roads, near markets, schools, offices, major crossroads and stopping points for “traditional” buses and taxis. They all have a park chief [30]. The experiment will be implemented among motorcycle taxi drivers in parks in Parakou (intervention group), while those in Porto Novo will not benefit from the awareness activity package and will be the control group.

### Type of study

**Fig. 2:**
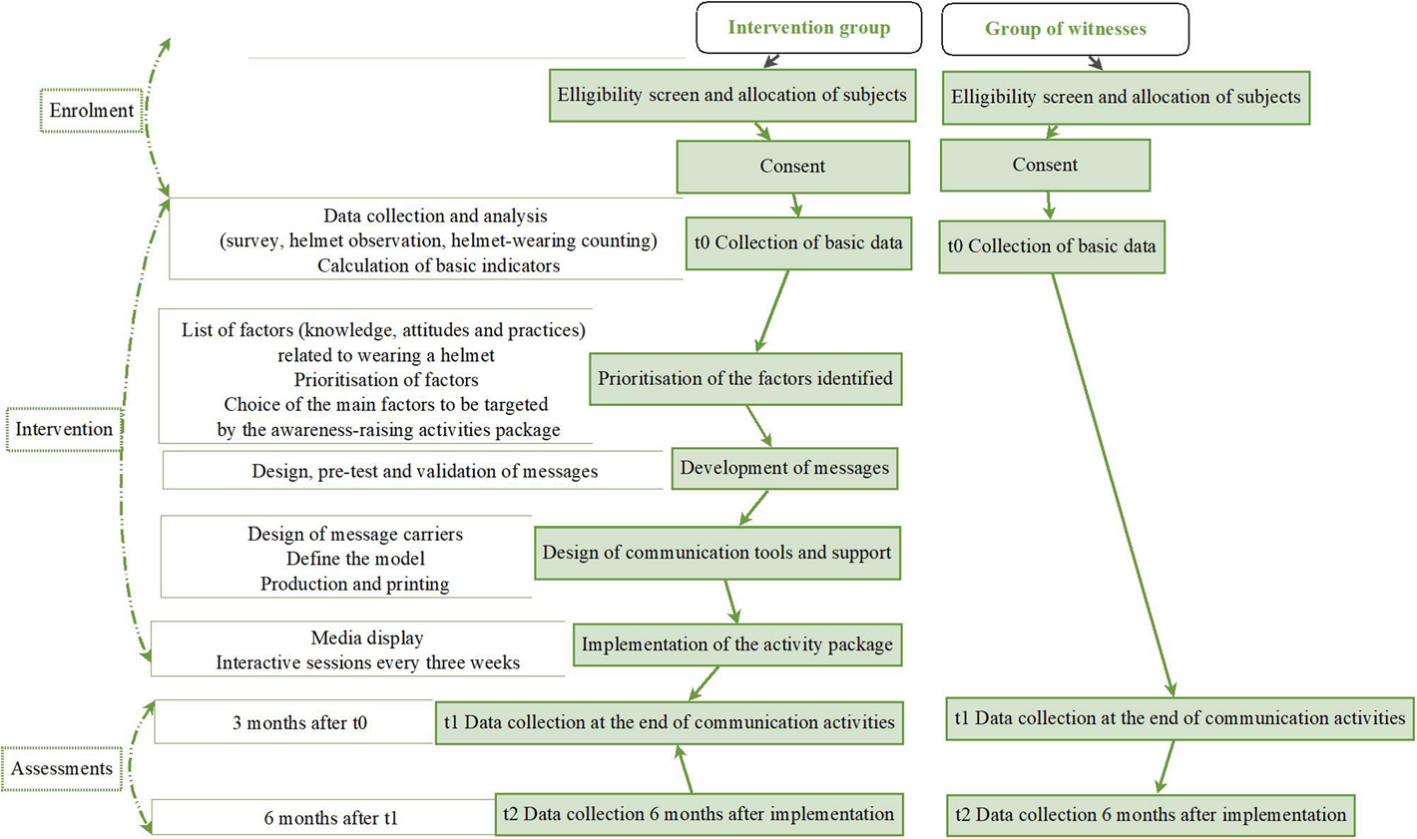
Overview of the study. In the green background are the different stages of the study in each group, and in the white background, the main activities at each stage and the timing

It is a quasi-experimental study that uses control groups and pre-tests [31], which will be conducted with motorcycle taxi drivers. Figure 2 shows an overview of the study scheme.

### Targets and inclusion criteria

The targets of the study will be two groups of motorcycle taxi drivers from Parakou and Porto Novo. The motorcycle taxi drivers in the Parakou group will receive the awareness activity package, and those from Porto-Novo will not. To be included in this study, motorcycle taxi drivers must be at least 18 years old, have frequented one of the selected parks regularly for at least 3 months, drive a motorcycle taxi as a main and daily activity, and be willing to participate in the study.

### Sampling

Sampling will be done at two stages. In each city, the list of the main parks will be obtained from the town hall. Two parks will be chosen at random from the parks in each city. In each park, the drivers will be informed in collaboration with the park managers. Within the parks, convenience sampling will be used. All drivers who meet the inclusion criteria will be recruited. The group of taxi-motorcyclists recruited in each city at the start of the study will form a closed cohort, from which the various data will be collected. In the study area, it is this cohort that will primarily benefit from the intervention.

### Sample size

The sample size will be calculated using the formula for determining the sample size to compare two groups when the outcome sought is qualitative n = (*Z*_α/2_ + Z_β_)^2^[p_2_(1 − p_2_) + p_1_(1 − p_1_)]/(p_2_ − p_1_)^2^ [32, 33]. The proportion p_2_ of subjects with good helmet-wearing behaviour before the start of the intervention (control group) is 63.1%. This is the proportion of respondents in Thika who reported that they always wore a helmet [10]. The minimum desired difference to highlight is 25%. Quine et al. obtained this difference as the proportion of school-age cyclists who used a helmet after an intervention based on the theory of planned behaviour [27]. The proportion p_1_ expected in this study (intervention group) will therefore be 88.1%. The risk α = 5%; z_α/2_ = 1.96. The power 1-β = 80%; z_β_ = 0.842; which gives a minimum of 42 for each zone (intervention, non-intervention). Application of the continuity correction ${n}^{\prime }=\frac{n}{4}\Big[1+\sqrt{1+\frac{4}{n\mid {\mathrm{p}}_2-{\mathrm{p}}_1\mid }}$ [33], and a lost-to-follow-up rate of 10%, leads us to estimate that at least 55 people will be recruited from each group.

### Intervention

This is the implementation of a package of awareness-raising activities in the intervention area, preceded by a series of preparatory activities, such as prioritising key factors, developing messages, and designing tools (Fig. 1). This package will supplement the helmet-wearing controls, penalties and mass awareness activities carried out in both areas.

#### Prioritisation of factors

During the baseline collection, data on helmet-wearing knowledge, attitudes and practices will be collected. Based on this collected data, the most frequently mentioned elements will be retained as individual factors to be targeted for change. The awareness-raising messages to be developed will focus on these factors.

#### Development of messages

The key messages and images to be disseminated through the various tools and communication channels will relate to the importance of wearing helmets to prevent traumatic brain injury. They will be developed after the baseline collection, based on those influencing factors identified in the process as needing to be reinforced or modified. Images/photos and videos will be selected from various sources decided by the design team. These messages and images will be validated with the support of a communications specialist, a communications officer from the National Centre for Road Safety (CNSR), staff from non-governmental organisations (NGOs) working on road safety in Benin (Alinagnon, and Young and Development), and a group of motorcycle taxi drivers. They will then be pre-tested with drivers from an area not involved in the study.

#### Communication tools design

Banners, motorcycle stickers and motorcycle taxi uniforms will be used to carry the validated key messages.

#### Implementation of the awareness-raising activities package

This will mainly involve local communication in the intervention area with interactive awareness sessions on helmet wearing for drivers, and the dissemination of messages through other channels.

- Interactive sessions: these will be discussion sessions, which will take place every 3 weeks on specific dates. Four sessions will be conducted during the awareness-raising period of approximately 3 months. They will be held in the parks in the intervention area. During the sessions, important messages will be communicated. Awareness images and videos will be shown to illustrate and reinforce the messages. At each session, the messages and images will be broadcast and rebroadcast for three to 5 h in order to reach the maximum number of motorcycle taxi drivers frequenting these parks. Interactive discussions around these messages will be held with the drivers to ensure a good understanding of the messages, and to clarify their concerns in order to encourage them to adopt acceptable helmet-wearing attitudes and practices. All those present in the parks on the day of the session will be able to benefit from the communicated messages.
- Banners: in each park in the intervention area, a large banner carrying an awareness-raising message will be posted at a strategic location throughout the duration of the awareness phase. All drivers and passengers who frequent these parks will be able to benefit from these messages.
- Stickers for motorcycles and motorcycle taxi uniforms, carrying validated messages, will be distributed to each driver included in the cohort at the start of the awareness phase. The stickers will be affixed directly to a clearly visible part of the motorcycles.

### Data collection

Data will be collected prospectively, before the implementation of the activities, at the end, and 6 months later.

#### Baseline data collection

This data collection will take place at t_0_ in September 2020, before the start of the awareness-raising phase in the two zones (intervention and control), in order to have an understanding of the baseline situation before the implementation of the awareness-raising activities. It will be in the form of a survey on knowledge, attitudes and practices (KAP) about helmet wearing. Analysis of the data from this initial collection will be used to identify the individual factors to be reinforced or changed, and around which the key messages will be developed during the awareness raising activities. A helmet-wearing count will also be made by observation, for comparison with the declarations and to assess the actual prevalence of helmet wearing. This collection will also provide the level of key indicators to be calculated during the study, to compare with those after the awareness-raising.

#### Data collection after implementation

Data will be collected at t_1_ at the end of the activity package (January) to assess the gains in awareness (immediate effects), and again at t_2_ 6 months later to assess maintenance of the awareness gains and the medium-term effects. The same data will be collected as in the baseline collection, using the same tools. Information about injured drivers will be completed via the self-reported questionnaire.

#### Data collection techniques and tools

**Fig. 3:**
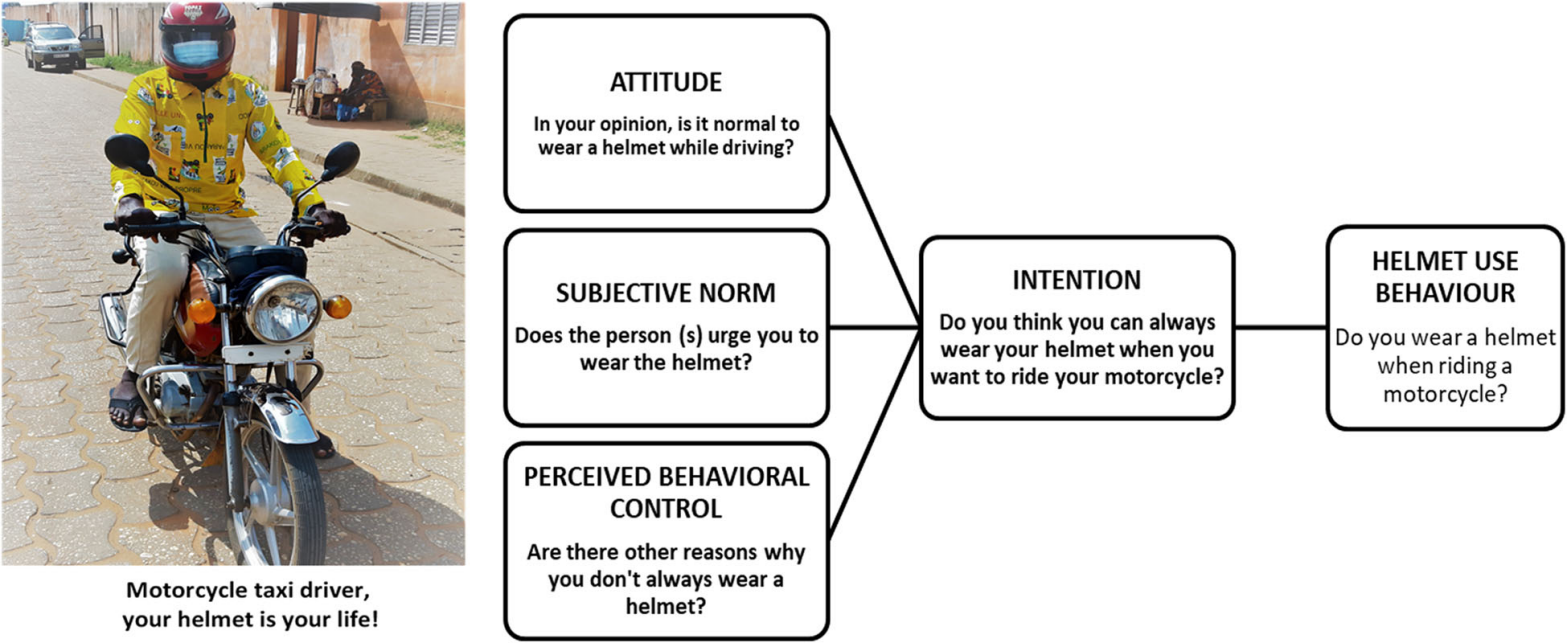
Illustration of a message to put on motorcycle taxi uniforms (left), and some examples of the questions formulated in the self-reported questionnaire, designed based on the theory of planned behaviour (right). Copyright holder: BHDS

A self-reported questionnaire with a structured survey and observation will be used for data collection. The questionnaire was designed based on the theory of planned behaviour. Figure 3 illustrates some examples of the questions formulated to assess each component of this theory and achieve the objectives of the study. Observation will be based on a helmet count entered in an observation grid. The questionnaire and the helmet observation grid will be configured on tablets using KoboCollect software. Input controls will be introduced when designing the tablet-based questionnaire.

#### Organisation of data collections

The tools will be pre-tested with motorcycle taxi drivers in an area not involved in the study. Investigators trained in the collection methodology, the use of tablets and the data entry will administer them. During the collection period, four investigators will be sent to each park. Two will be responsible for counting the number of helmets worn. The other two will be responsible for surveying the motorcycle taxi drivers, i.e. administering the questionnaire and completing the helmet observation grid. The two counters will be outside the park, a short distance apart, on either side of the entrance. They will double count the number of helmets worn, for comparison with the observed data at the end of the day. For the administration of the questionnaire, a corner will be found away from other motorcycle taxi drivers. Investigators must be fluent in French, in addition to the local language most spoken in the locality. They will administer the questionnaire in French or in the local language, depending on the motorcycle taxi driver. One team leader per study area will supervise the interviewers and ensure the quality of the interviews and the data entry. In addition to the actions directly related to the study, and to contribute to the fight against Covid-19, all the riders in the parks visited will also benefit from group awareness-raising on Covid-19 with an emphasis on respect for health prevention measures.

#### Data to be collected and indicators for measuring results

The data to be collected will relate to:

- General Information:
- Socio-demographic data (age, sex, marital status, ethnicity, religion, level of education, average income, number of dependents).
- History (how long they have been driving motorcycles and in the motorcycle taxi profession, whether they own their motorcycle, road accidents, number of days of driving per week, average number of hours of driving per day, sanctions for not wearing a helmet).
- Knowledge: Five (5) questions (advantages, disadvantages, characteristics of a quality helmet).
- Attitudes: Eight (8) questions (perception, judgement related to wearing a helmet).
- Subjective norms: Four (4) questions (influence of those around you).
- Perceived behavioural control: Three (3) questions (perceived constraints in relation to wearing a helmet).
- Intention of wearing a helmet: Four (4) questions (possession of a helmet and reason for purchase, willingness to wear helmet).
- Practices of use, and information on the helmet: Six (6) questions (frequency, time/period of wearing of the helmet, mode of use, type and condition of the helmet).
- The occurrence of a road accident and traumatic brain injury.

### Data processing and analysis

The data collected via KoboCollect will be extracted and processed using Excel and Stata 15 software. They will be analysed using Stata 15. Identifiers will be given to each subject enrolled in the database. With these identifiers, the data of each subject for different collections will be linked for the analysis. The initial postulates are that:

- The two groups are similar at the start of the intervention and that only the fact that one group benefits from the experiment differentiates them.
- The proportion of changes due to factors other than the awareness-raising activities carried out as part of the intervention does not differ between the two groups (intervention group and control group).
- The difference between the two cities at the end of the intervention can be considered to have been due to the awareness-raising activities carried out.

When analysing the baseline data, the study population will be described according to their socio-demographic data and the number of interactive sessions in which they have participated. The subjects included in the initial data collection, but who do not respond to the other collections, will be compared with the respondents in order to verify the existence of a bias. These comparisons will be made using the Chi^2^ test after checking that the conditions are met (the expected values ≥5). If the conditions are not met, we will use Fisher’s exact test.

The actual data analysis will compare subjects not excluded from the intervention group with those from the control group. An overall score for level of knowledge, attitude and practice will be calculated for each individual. This overall score will be obtained from the scores of the different groups of variables (knowledge, attitudes and practices). Scores will be calculated by assigning points to each response given by the enrolled subject. The total points will be calculated to keep the score for each group of variables. These scores will vary as follows, by group of variable: knowledge (0 to 14), attitudes (0 to 24), subjective norms (2 to 13), perceived behaviour control (0 to 1), intention (1 to 17) and practices (0 to 28). The scores obtained by each individual will be compared before and after the intervention. Student’s statistical test for paired series will be used for these intra-group score comparisons. For these tests, the equality of variances will be tested using the robust Levenne’s test for variance of equality. If this test is significant, the Hartley test (S^2^max/S^2^min < 3) will be performed. If the variances and distribution are not respected, a non-parametric test will be used (Kruskal-Wallis test).

Average or median scores will be calculated by zone (intervention and control) for each collection. Comparisons will also be made between the mean scores of pre- and post-awareness, intervention and control areas, and according to socio-demographic characteristics. Student’s statistical test or non-parametric tests will be used for these comparisons.

The proportion of motorcycle taxi drivers whose score increases will be compared by area and by period. For the comparison of the proportions, the Chi^2^ test will be used after checking that the conditions are met (the expected values ≥5). If the conditions are not met, we will use Fisher’s exact test.

After this preliminary analysis, the difference-in-difference (DD) estimator, an approach using a linear parametric model, will be used [34] to determine the specific effect of the awareness-raising activities in order to assess whether these have brought any added value. This estimator is the difference in mean overall score in the intervention group before and after the awareness-raising activities, from which the same difference is subtracted in the control group. It corresponds to the coefficient β_3_ of the regression equation *Y*_*i*_ = *β*_0_ + *β*_1_*T*_*i*_ + *β*_2_*t*_*i*_ + *β*_3_(*T*_*i*_ ∗ *t*_*i*_) + *λX*_*it*_ + *ε*_*i*_ in which *Y*_*i*_ is the overall score of the subjects, *T*_*i*_ the groups (intervention and control), *t*_*i*_ the period (pre- and post-intervention), *X*_*it*_ the variables related to the socio-demographic characteristics and background of the subjects, and *ε*_*i*_ the random error.

The significance level of the statistical tests will be 5%.

## Discussion

The subjects present in the parks on the data collection days, and/or those who consent, may not be representative of the overall population of motorcycle taxi drivers. Convenience sampling could therefore generate a risk of selection bias. This risk could be reduced by minimising the cases of refusal and by enrolling almost all of those present in the parks on the collection days. This study will be based on the implementation of an intervention in motorcycle taxi parks in order to assess the contribution of awareness messages to changing drivers’ behaviour. To affect behaviour change, it will be important to develop quality messages that are well understood by the targets. For this purpose, various road safety stakeholders and some motorcycle taxi drivers will be involved in the design of the messages. A second challenge will be to reach all the motorcycle taxi drivers included in the intervention group with all the awareness-raising activities. Finally, successfully maintaining all of these subjects in the cohort, both in the intervention group and in the control group, will also be a major challenge. To meet this challenge, the phone numbers of the enrolled subjects will be taken, and they will be contacted before each interactive session and each future data collection. The targets of the study are motorcycle taxi drivers waiting for customers in parks. As such, they could be more concerned with finding customers during awareness campaigns, which would reduce their acquisition of the message and its effect. In addition, contextual elements could influence the results obtained.

As research in the field of road accident prevention is rare in Benin, this study will help fill a significant gap. It will provide factual data on the rate of helmet use among motorcycle taxi drivers and on their KAP relating to helmet use.

## Data Availability

The data and materials for this study will be available from the corresponding author. To access this data, contact the main author.

## Abbreviations

CNSR: National centre for road safety
DD: Difference in difference
KAP: Knowledge, attitudes and practices
NGO: Non-governmental organisations
WHO: World health Organization
